# Histological, Immunohistological, and Clinical Features of Merkel Cell Carcinoma in Correlation to Merkel Cell Polyomavirus Status

**DOI:** 10.1155/2012/983421

**Published:** 2012-05-07

**Authors:** T. Jaeger, J. Ring, C. Andres

**Affiliations:** Department of Dermatology and Allergy Biederstein, Technische Universität München, 80802 Munich, Germany

## Abstract

Merkel cell carcinoma is a rare, but highly malignant tumor of the skin with high rates of metastasis and poor survival. Its incidence rate rises and is currently about 0.6/100000/year. Clinical differential diagnoses include basal cell carcinoma, cyst, amelanotic melanoma, lymphoma and atypical fibroxanthoma. In this review article clinical, histopathological and immunhistochemical features of Merkel cell carcinoma are reported. In addition, the role of Merkel cell polyomavirus is discussed.

## 1. Introduction

Merkel cell carcinoma (MCC) is one of the most malignant tumors of the skin which affects primarily sun-exposed skin from older Caucasian, predominantly males with a mean age at the time of diagnosis of about 70 years [1]. Its incidence rate rises with immunosuppression [2, 3], and is currently 0.6/100000/year [4]. Its biological behavior is highly aggressive with high rates of metastasis and poor survival [1]. If the Merkel cell is the cell of origin of this cancer is still matter of debate. Recently, it was reported that Merkel cells are derived from the epithelial lineage [5]. Besides, because of sarcomatous elements were found in MCC, so a totipotent epithelial stem cell as origin was suggested [6], but further examinations are requested.

## 2. Clinical Features

MCC characteristically develops rapidly and asymptomatically over months [7]. Most MCCs are located on sun-exposed areas. About 50% of MCCs occur on the head and neck, 40% on the extremities and remainder on the trunk and genitalia [8]. It very rarely arises on sun-protected areas, such as the oral and genital mucous membranes, where it is characterized by a particularly poor prognosis [9, 10]. It usually manifests as solitary, firm, flesh-colored to red nodule with a smooth, shiny surface, sometimes with telangiectasia [11, 12]. Differential diagnosis includes basal cell carcinoma, cyst, amelanotic melanoma, lymphoma, and atypical fibroxanthoma [13].

The five most common clinical features were used to create an acronym *AEIOU* [14];

Asymptomatic/lack of tenderness,
Expanding rapidly (≤3 months),
Immunosuppression,
Older than age 50,
UV-exposed site.

## 3. Histopathology

MCC usually appears as a dermal tumor nodule, which frequently extends into the subcutaneous fatty tissue. The tumor cells are small blue cells with basophilic nuclei and minimal cytoplasm. Mitoses are frequent and the apoptosis index is high [15]. The papillary dermis and adnexa are usually spared [16].

**Figure 1:**
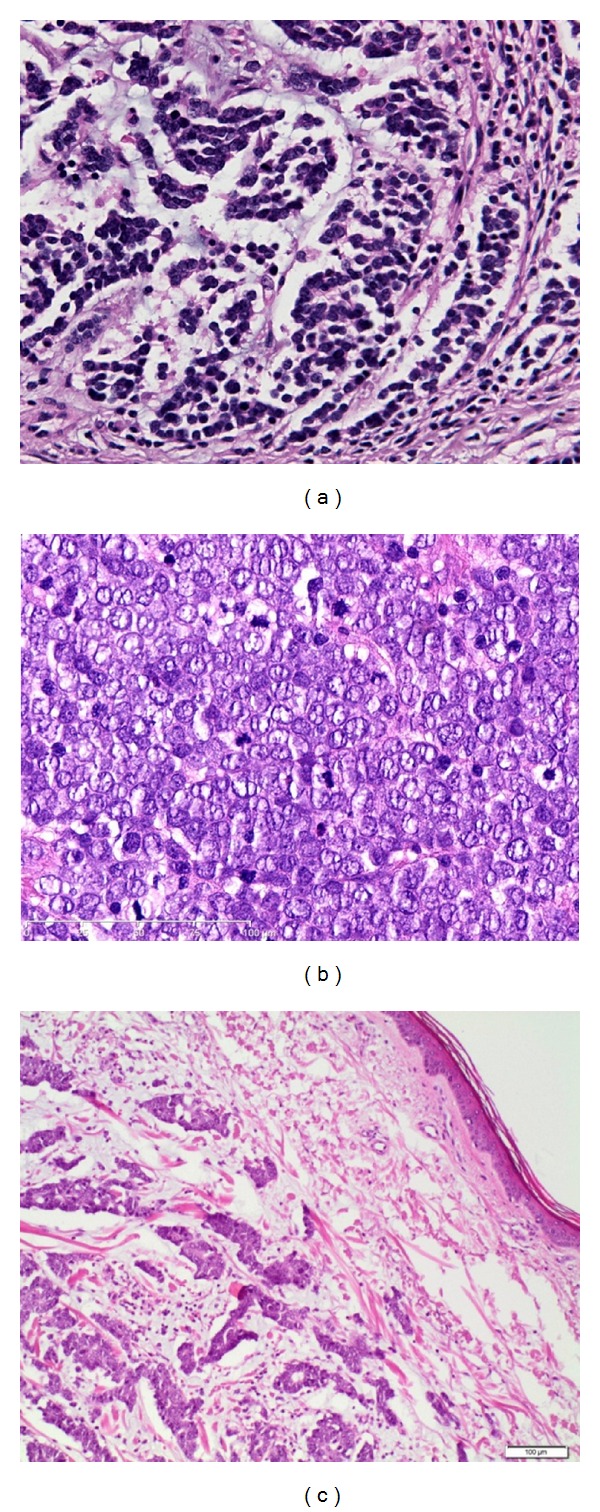
(a) Small-cell variant, histologically indistinguishable from bronchial small-cell carcinoma. (b) Intermediate variant of MCC showing vesicular, basophilic nuclei with prominent nucleoli and high mitotic activity. (c) Trabecular variant is rare and normally only seen as a small component of a mixed variant.

Three histologic subtypes (showen by Figure 1) have been recognized [4]:

- the intermediate type,
- the small cell type,
- the trabecular type.

In the latest data the trabecular form is discussed as the best differentiated with a better prognosis, while the small cell form is relatively undifferentiated and has a worse prognosis. But comprehensive data are missing and mixed and transitional forms are frequent, so there is no clear histologic-prognostic association. 

Tumor size ≤ 2 cm, female gender, primary tumour localized at the upper limb, and pathologically proven negative lymph nodes are factors highly significant for prognosis and are incorporated into the new staging system for MCC [6, 19].

Andea et al. evaluated retrospectively the following histologic features with regard to prognosis: tumor thickness, microanatomic compartment involved by tumor (dermis and/or subcutis and/or deeper), tumor growth pattern (nodular circumscribed versus infiltrative), lymphovascular invasion, tumor-infiltrating lymphocytes, tumor necrosis, ulceration, and solar elastosis. On multivariate analysis, tumor thickness, the presence of a nodular growth pattern, low tumor depth and absence of lymphovascular invasion were statistically significantly associated with longer survival [20].

Morphometric analyses revealed that Merkel cell polyomavirus-negative Merkel cell carcinomas show a different histologic appearance with more irregular nuclei and more abundant cytoplasm than Merkel cell polyomavirus-positive Merkel cell carcinomas, which are characterized by uniform round nuclei and scant cytoplasm [21].

## 4. Immunohistochemistry

The “small round blue cell” histologic pattern of MCC must be differentiated from several other tumors, such as small-cell lung carcinoma, carcinoid tumor, malignant lymphoma, and small-cell melanoma. Therefore immunohistochemical stainings are required. MCCs are positive for epithelial and neuroendocrine markers, but are negative for lymphoid and melanoma markers [15]. Table 1 shows characteristic immunohistochemical staining patterns for these entities.

Positive staining for CK20 and NSE are quite specific for MCC. Anti-cytokeratin 20 (CK20) staining is concordant to data from the previous literature showing “paranuclear dot-like pattern” in 97% of all included MCCs [7]. This highly sensitive staining feature is very important for routine histopathology to distinguish MCCs from other small round blue cell tumors [22, 23].

Thyroid transcription factor-1 (TTF-1) is usually expressed in small-cell lung carcinoma but is consistently absent in MCC [7]. Leucocyte common antigen (LCA) is negative in MCC, but positive in lymphoma [18, 24]. Small cell carcinoma of the lung (SCLC) is cytokeratin 7 (CK7) positive, but not MCC [22].

Another useful marker for the distinction between MCC and small-cell lung carcinoma is the neurofilament protein (NFP), which is usually positive in MCC and always negative in small-cell lung carcinoma [22].

The differentiation between MCC and malignant melanoma is based on the negativity of the latter for CK 20 and its positivity for HBM45, NKI/C3, and S-100, for which MCC is usually negative [25].

Further, the tumor cells of MCC display additional antigens in varying frequency and intensity; these include, among others, chromogranin A, synaptophysin, tenascin-C, CD56 as well as various neurofilaments and neuropeptides. Expression of the inhibitor of apoptosis (IAP) survivin and the member of the p53 family p63 appears to be associated with a poorer prognosis [17, 26–28].

## 5. Merkel Cell Polyomavirus

Although MCC is one of the most aggressive skin cancers with a high mortality rate, little is known about potential signalling mechanism that drives carcinogenesis in MCC. The association of MCC with immunosuppression has prompted the hypothesis of a viral implication in the pathogenesis of the tumor. But the published data on the impact of Merkel cell polyomavirus (MCPyV) presence or viral load on prognosis remain controversial.

Merkel cell Polyomavirus (MCPyV) was identified in January 2008 by Feng and colleagues in tumor tissue from MCC patients, proving clonal integration of the virus DNA into the host genome [25]. Meanwhile several studies confirmed this observation showing frequent prevalence of MCPyV DNA in MCCs [29–35]. These data suggest MCPyV as the likely causative agent of MCC. It is supposed that an interaction of the MCC virus protein with p53 and members of the retinoblastoma (Rb-) Gen family could be responsible for the malignant degradation [30, 36].

Furthermore, Sihto et al. found evidence for better prognosis in MCPyV DNA-positive MCC having fewer regional nodal metastasis at time of diagnosis compared to MCPyV DNA-negative MCC [34]. Andres et al. showed that MCPyV DNA-positive MCCs tend to be preferentially located on the limbs and tend to metastasize less frequently [37]. It seems that MCPyV MCCs harbour more genomic aberrations than MCPyV ones [38]. Besides it has been reported that absence of MCPyV or lower viral abundance is associated with increased p53 and KIT espression [39, 40]. Meanwhile, latest data did not show better clinical prognosis in patients with MCPyV-positive MCCs [2, 25].

## 6. CK20-, CK19-, CD117-, and ST-3 Protein Expression of Tumor Cells as a Function of Presence of MCPyV DNA in MCC

There is only one report analyzing immunohistochemical features of MCCs in correlation to presence of MCPyV DNA [41]. In the cohort studied there is no statistical significant association between MCPyV DNA prevalence and immunohistochemical expression of CK20, CK19, CD117, and ST3 was detected but some exciting trends. 

CK 19 is a small human cytokeratin, expressed in undifferentiated germinative basaloid cells and usually not expressed by cells of nonepithelial origin [42]. CD117 is a transmembrane protein of the receptor tyrosine kinase family. Stromeylsin-3/matrix metalloproteinase11 (ST3) overexpression could be associated with tumor invasion because of a antiapoptotic effects [43]. A more frequent CK19 expression in MCPyV DNA-negative MCCs and CD117 expression in MCPyV DNA-positive MCCs was observed. Moreover, CK19 is a helpful diagnostic marker for CK20-negative MCC. The role of ST3 expression is not yet clear, being expressed by the MCC tumor cells themselves in about half of all cases, independent of MCPyV DNA-prevalence. However, in some studies MCPyV DNA-prevalence seems to influence the biological behavior of MCCs, resulting in better overall survival for patients with positive MCPyV DNA-status [34]. Most probably due to different invasion and metastatic properties, Andres et al. were not able to find statistically significant differences in the expression pattern of CK20, CK19, CD117, and ST3 [41].

In conclusion, more data will be needed to get profound insight in the carcinogenesis of MCC. As MCC is a rare cancer, studies are limited and further molecular studies are required as well as clinical investigations to establish the impact of MCPyV on MCC which perhaps could open new therapetic options.

## Figures and Tables

**Table 1:** Immunohistochemistry of Merkel cell carcinoma (according to Schrama et al. 2011, Becker et al. 2008; [17, 18]). CK20: cytokeratin 20; CK7: cytokeratin 7; NSE: neuron-specific enolase; TTF1: thyorid transcriptor factor 1; LCA: leucocyte common antigen.

	CK20	CK7	NSE	TTF1	S100	LCA
Merkel cell carcinoma (MCC)	**+**	−	**+**	−	−	−
Small cell carcinoma of the lung (SCLC)	−	**+**	**+**	**+**	−	−
Melanoma	−	−	−	−	**+**	−
Lymphoma	−	−	−	−	−	**+**

## Abbreviations

CK7: Cytokeratin 7
CK 20: Cytokeratin 20
LCA: Leucocyte common antigen
MCC: Merkel cell carcinoma
MCPyV: Merkel cell Polyomavirus
NSE: Neuron-specific enolase
TTF-1: Thyroid transcription factor-1
SCLC: Small cell carcinoma of the lung.
